# p53: A Key Protein That Regulates Pulmonary Fibrosis

**DOI:** 10.1155/2020/6635794

**Published:** 2020-11-29

**Authors:** Qi Wu, Ke-jia Zhang, Shi-min Jiang, Lu Fu, Yue Shi, Ru-bin Tan, Jie Cui, Yao Zhou

**Affiliations:** ^1^Department of Physiology, Xuzhou Medical University, Xuzhou 221009, China; ^2^Department of Pathophysiology, Xuzhou Medical University, Xuzhou 221009, China; ^3^Laboratory of Clinical and Experimental Pathology, Xuzhou Medical University, Xuzhou 221009, China

## Abstract

Pulmonary fibrosis is a progressively aggravating lethal disease that is a serious public health concern. Although the incidence of this disease is increasing, there is a lack of effective therapies. In recent years, the pathogenesis of pulmonary fibrosis has become a research hotspot. p53 is a tumor suppressor gene with crucial roles in cell cycle, apoptosis, tumorigenesis, and malignant transformation. Previous studies on p53 have predominantly focused on its role in neoplastic disease. Following in-depth investigation, several studies have linked it to pulmonary fibrosis. This review covers the association between p53 and pulmonary fibrosis, with the aim of providing novel ideas to improve the clinical diagnosis, treatment, and prognosis of pulmonary fibrosis.

## 1. Introduction

Pulmonary fibrosis is a group of chronic, irreversible, and fatal interstitial lung diseases that occur mostly in middle-aged and elderly people [1]. It mainly presents as fibrosis and honeycomb changes of the subpleural and basement membranes, as well as the deposition of collagen and extracellular matrix (ECM) around the fibrotic foci. Pulmonary fibrosis ultimately leads to life-threatening structural changes in lung tissue and loss of pulmonary ventilation and diffusion [2]. The most common type of pulmonary fibrosis is idiopathic pulmonary fibrosis (IPF). The median survival for IPF is only 2-4 years [3]. Thus, it is a serious lung disease that threatens human health. The incidence of pulmonary fibrosis is increasing with the aging of the population. Unfortunately, the pathogenesis of pulmonary fibrosis is still poorly understood and there are no effective therapeutic drugs [4]. Therefore, it is important to elucidate the pathogenesis of pulmonary fibrosis and identify suitable therapeutic drugs.

**Figure 1:**
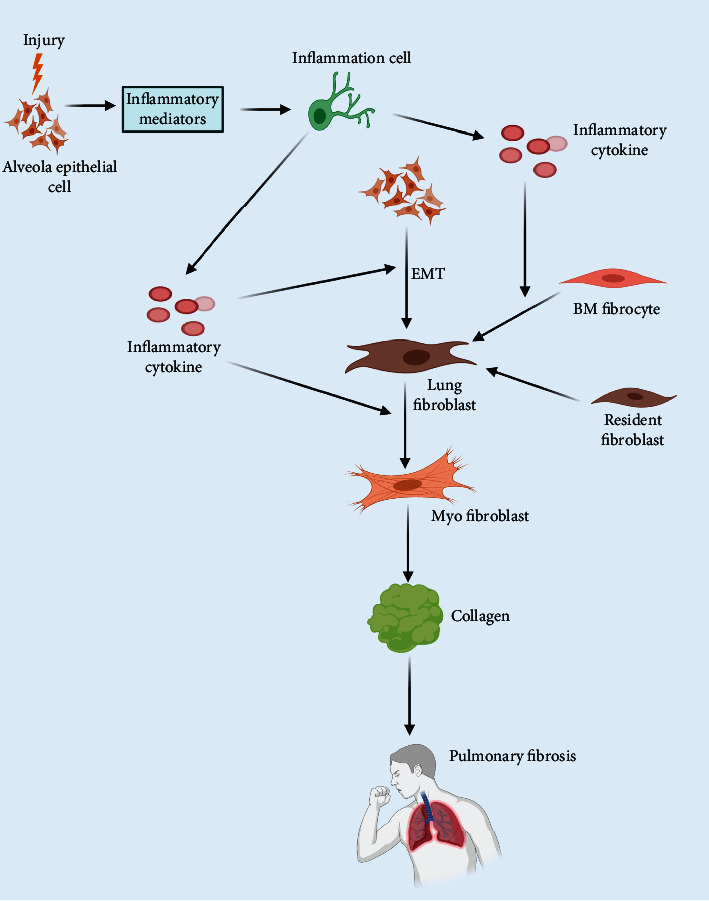
Pathogenesis of pulmonary fibrosis. The pathogenesis of pulmonary fibrosis originates after damage to the alveolar epithelial cells (AECs). Damaged AECs can induce the relevant inflammatory cells to secrete inflammatory factors. These inflammatory factors can stimulate the transition of bone marrow (BM) fibrocytes to lung fibroblasts, accelerate the EMT process, induce the transition of AECs to lung fibroblasts, and promote the transition of lung fibroblasts to myofibroblasts. Myofibroblasts can secrete a large amount of collagen, which further accelerates the progression of pulmonary fibrosis.

It is currently accepted that the development and progression of pulmonary fibrosis are attributable to aberrant repair following repeated alveolar epithelial cell (AEC) injuries in response to various stimuli [5]. The injured alveolar epithelial cells (AECs) can secrete various cytokines, such as transforming growth factor-*β*1 (TGF-*β*1), tumor necrosis factor-*α* (TNF-*α*), and platelet-derived growth factor (PDGF) [6–8]. These cytokines may facilitate the development of a fibroblast focus through multiple pathways, such as pulmonary epithelial-mesenchymal transformation (EMT), the proliferation of mesenchymal cells, and the recruitment of cycling fibroblasts [9]. This leads to mass deposition of collagens and eventually affects the normal structure and function of lung tissue [10] (Figure 1).

For a long time, the function of the inflammatory response in pulmonary fibrosis remained debatable. Conventionally, pulmonary fibrosis is believed to be a chronic inflammation-related response [11]. Inflammatory responses are induced mediated by alveolar epithelial cells in response to injury [12]. Inflammatory factors can act directly on pulmonary alveoli; this may aggravate the injury, but may also form part of a vicious cycle through the activation of relevant inflammatory cells and lymphocytes [13] (Figure 1). However, an inflammatory response is probably not necessary for the development and progression of pulmonary fibrosis. It may only occur in the early stage of pulmonary fibrosis [14]. However, evidence supporting the involvement of the inflammatory response in pulmonary fibrosis is still lacking. The mechanisms of injury and maladjusted repair during pulmonary fibrosis have not been defined. Therefore, it is too early to draw the conclusion that the inflammatory response does not participate in the genesis and development of pulmonary fibrosis.

During the development and progression of pulmonary fibrosis, the protection of AECs, inhibition of EMT, removal of lung fibroblasts, inhibition of lung fibroblast proliferation and collagen secretion, and the alleviation of inflammatory reactions are crucial to retard the progression of pulmonary fibrosis [15–17]. In-depth studies have revealed multiple proteins and signaling pathways that are dysregulated in pulmonary fibrosis. According to recent findings, p53 is believed to play a pivotal role in the development and progression of pulmonary fibrosis through the modulation of apoptosis, aging, oxidative stress, EMT, and other cellular processes. This review discusses the role of p53 in pulmonary fibrosis.

## 2. p53

The p53 gene is an important tumor suppressor gene, located on human chromosome 17P13.1, which consists of 11 exons and 10 introns. Since it was first discovered in 1979, this gene has been a research “hotspot” [18]. The p53 protein monomer contains 393 amino acid residues [19]. The N-terminus contains a transactivation domain (TAD) and a proline-rich domain (PRD), and the C terminus (CT) is a free unfolding domain that can bind to DNA in an unspecific manner [20]. The two termini are connected by a core DNA-binding domain and a relatively short tetramerization domain (TD) [21]. The wild-type p53 gene can undergo several mutations, including point mutation, deletion, frameshift, and rearrangement [22–24]. Detection of the wild-type p53 protein is difficult owing to its short half-life [25]. The spatial conformations of mutant p53 proteins differ, and they have longer half-lives. Therefore, they can be observed using immunohistochemical methods.

The p53 gene is one of the most important and well-studied tumor suppressor genes to date [26]. Owing to its critical role in normal cell growth, inhibition of malignant tumor growth, and regulation of the cell cycle, p53 is usually considered a guard gene [27]. p53 is activated in response to various stimuli and can be attuned to stress in a transcription-dependent or -independent manner [28]. Through extensive research, p53 was found to play a critical role, not only in cancer, but also in the regulation of pulmonary fibrosis.

## 3. Relationship between p53 and Pulmonary Fibrosis

In one study, researchers collected the lung tissues of 10 patients with pulmonary fibrosis and examined p53 for single-strand conformation polymorphism (SSCP). Nine point mutations were found in ten lung tissue samples. Most of the mutations occurred in the central area of the p53 gene [29]. In another study, researchers collected lung tissues from 14 patients with idiopathic pulmonary fibrosis (IPF) and 19 healthy individuals. They found that the p53 gene was highly expressed in the lung tissues of patients with IPF, and the main form observed was the wild-type p53 gene. It is therefore speculated that the enhancement of the wild-type p53 gene is probably a compensatory response of the body to injury. The p53 protein may prevent the proliferation of injured or aberrant cells and alleviate lung tissue injury by regulating the cell cycle [30]. Korthagen et al. analyzed single nucleotide polymorphisms (SNPs) in the p53 gene from the lung tissues of 66 patients with IPF and 353 healthy controls and found that rs12951053 and rs12602273 were significantly correlated with the survival of patients with IPF. The four-year survival rate of carriers was 22% and that of noncarriers was 57% [31].

Some research studies have addressed the relationship between p53 and pulmonary fibrosis. Of the numerous methods to produce in vivo models of pulmonary fibrosis, the most common method is the injection of drugs, including bleomycin (BLM), amiodarone, and asbestos [32]. A single intratracheal instillation of BLM is the most popular. Initially, this administration damages AECs, and these damaged cells release a variety of inflammatory factors, including TGF-*β*1, to accelerate the EMT process and the proliferation of lung fibroblasts. This leads to the secretion of more collagen, which further accelerates pulmonary fibrosis. Many preliminary studies of pulmonary fibrosis have utilized this model [33, 34]. Following the intratracheal injection of BLM in mice, a successful pulmonary fibrosis model was established after 28 days, and it was found that the p53 protein expression was significantly elevated in lung tissues of these mice [35]. In other studies, mouse models of pulmonary fibrosis were established through the intratracheal injection of BLM into WT and p53-deficient mice. These studies reported significant reductions in the amount of lung tissue damage and collagen deposition in p53-deficient mice compared with that in the wild type (WT) mice [36]. This finding suggests that inhibition of p53 expression can slow down the progression of pulmonary fibrosis.

## 4. Potential Mechanisms Involved in p53-Mediated Regulation of Pulmonary Fibrosis

### 4.1. Cell Apoptosis

Cell apoptosis is a type of programmed cell death and a key process that regulates homeostasis at the organizational, tissue, and internal environment level [37]. There are currently three pathways for cell apoptosis: the death receptor apoptosis pathway, the mitochondrial apoptosis pathway, and the endoplasmic reticulum apoptosis pathway [38]. Although these three apoptosis pathways are not exactly the same, apoptosis is ultimately completed by cysteinyl aspartate proteinase-related proteins [39]. It has been proven that excessive alveolar epithelial cell apoptosis occurs during the development and progression of pulmonary fibrosis, and during this stage, lung fibroblasts have a major function in apoptosis resistance [40, 41]. Therefore, the alleviation of AEC apoptosis or the enhancement of lung fibroblast apoptosis can effectively reduce the degree of pulmonary fibrosis (Table 1).

**Figure 2:**
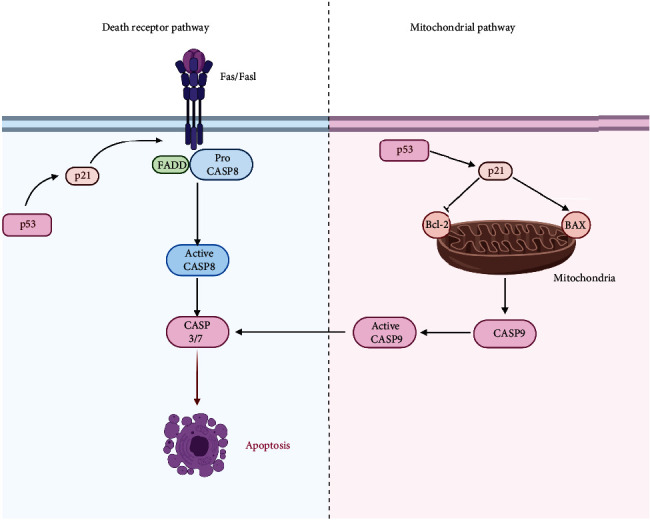
p53 promotes pulmonary fibrosis by inducing AEC apoptosis. p53-mediated AEC apoptosis is mainly involved in the death receptor apoptosis pathway and membrane receptor apoptosis pathway. In the death receptor apoptosis pathway, p53 activates p21, which then acts on Fas/Fasl to activate caspase-8 (CASP8) and initiate the death receptor apoptosis pathway. In the mitochondrial apoptosis pathway, p53 activates p21, which then promotes the expression of the apoptosis-related protein BAX to inhibit the expression of the antiapoptosis protein Bcl-2, and finally initiates the mitochondrial apoptosis pathway. Activation of either of these two apoptosis pathways leads to the activation of caspase-3/7 (CASP3/7) to promote cell death.

Researchers have constructed a mouse model of pulmonary fibrosis using a one-off intratracheal instillation of BLM. The results showed that apoptosis of type II AECs in the lung tissues of mice with pulmonary fibrosis was clearly enhanced, whereas the expression of the p53 protein was markedly increased. In contrast, knockout of the p53 protein effectively attenuated pulmonary fibrosis, and the extent of type II alveolar epithelial cell (AECII) apoptosis was also markedly alleviated, suggesting that the p53 protein probably accelerated the development and progression of pulmonary fibrosis by inducing AEC apoptosis [42]. In a clinical study that performed TdT-mediated dUTP Nick-End Labeling (TUNEL) staining of tissues, AEC apoptosis was markedly enhanced in the lung tissues of patients with IPF compared with the lung tissues of normal controls. Further study indicated that the expression of the p53 protein and apoptosis-related proteins caspase-3 (CASP3) and Bax was increased significantly and that of the antiapoptotic protein Bcl-2 was reduced substantially. This suggested that the p53 protein promoted AEC apoptosis during pulmonary fibrosis, which is presumably related to p53-mediated mitochondrial apoptosis [43]. Recently, the p21 protein was discovered. The p21 protein is an important member of the cyclin-dependent kinase inhibitor family [44]. The p21 protein has two main functional domains: a C-terminal PCNA-binding domain and an N-terminal CDK-cyclin inhibitory domain. PCNA can bind to DNA polymerase *δ* and many other proteins that are involved in DNA synthesis to promote DNA synthesis. p21 directly inhibits DNA synthesis by competing with PCNA to bind with other DNA synthesis proteins [45]. Many studies have demonstrated the important regulatory role of p21 in the origin and development of pulmonary fibrosis as a key downstream protein of p53. The successful regulation of apoptosis and cellular senescence by p53 often requires the involvement of p21. It was also found that AEC apoptosis and the expression of the p53 protein and the p21 protein in lung tissues of patients with IPF were markedly increased compared with the normal lung tissues. It was further confirmed that this process was probably related to Fas/Fasl-mediated receptor apoptosis [46]. The aforementioned studies suggested that the effect of the p53 protein on AEC apoptosis during the development and progression of pulmonary fibrosis was probably involved in the mitochondrial and death receptor apoptosis pathways (Figure 2).

In addition, some studies have documented that the p53 protein could induce apoptosis in lung fibroblasts and myofibroblasts during the progression of pulmonary fibrosis, which may further delay the progression of pulmonary fibrosis. The effects of astaxanthin on myofibroblast apoptosis were also studied. The results indicated that astaxanthin could induce myofibroblast apoptosis through the activation of the mitochondrial apoptosis pathway. Further studies have confirmed the critical role of the p53 protein in this process [47]. In another study, it was found that gallic acid could significantly induce apoptosis in lung fibroblasts isolated from mice with pulmonary fibrosis. It was further assumed that this process was probably related to the p53-mediated mitochondrial and death receptor apoptosis pathways [48].

### 4.2. Cell Aging

Aging is an inhibited state of cell proliferation, in which the cell cycle is arrested in phase G0 or G1 [49]. Morphologically, the cells are tabular with augmented nuclei and aggregated chromatin [50]. Models of senescence can be established in several ways. The most common approaches involve pharmacological interventions at the cellular level, in animals. The most common induction drugs are the chemotherapeutic drugs cisplatin and galactose. Detection of senescence relies on indicators such as *β*-galactosidase (*β*-gal), telomeres and telomerase, senescence-related heterochromatin foci, and senescence-associated secretory phenotype (SASP). Among these, *β*-gal is detected earliest and used most commonly as a senescence marker. This lysosome-derived enzyme increases lysosomal biosynthesis in senescent cells [51–53]. It is a specific marker of cell senescence.

Recent studies have shown that the aging-related secretory phenotype, telomere injury, epigenetic changes, mitochondrial autophagy injury, and other pathological processes are probably involved in the occurrence and development of cell aging [54]. In an in-depth study, it was found that cell aging plays an important role in pulmonary fibrosis, which mainly includes AEC aging and lung fibroblast aging. For AECs, a majority of studies showed that with the progression of pulmonary fibrosis, AEC aging becomes aggravated, leading to cell cycle arrest, the loss of AEC barrier function, activation and proliferation of fibroblasts, collagen deposition, and scar formation [55, 56]. During the progression of pulmonary fibrosis, lung fibroblasts age and secrete many cytokines, which promote the conversion of AECs to lung fibroblasts on one hand, and lung fibroblasts to myofibroblasts [57, 58]. Consequently, collagen secretion increases, and the progression of pulmonary fibrosis is accelerated. Therefore, the focus of drug research for the treatment of pulmonary fibrosis has concentrated on the delay of AEC aging and lung fibroblast aging (Table 2).

A mouse model of pulmonary fibrosis was constructed by the administration of BLM to investigate the correlation between p53 and collagen deposition. The results showed that collagen deposition and p53 protein expression in BLM-induced mice was significantly enhanced compared with that in WT mice. To confirm the mechanism underlying AEC aging, A549 cells were treated with BLM. It was found that the expression of the aging-related marker *β*-galactosidase in A549 cells was markedly enhanced, and that of the p53 protein was also clearly increased in a concentration-dependent manner, suggesting that p53 promoted AEC aging and accelerated the progression of pulmonary fibrosis [59]. It is reported that during the progression of pulmonary fibrosis, p53-mediated AEC aging was probably realized through the activation of the p21 protein. Upon activation by the p53 protein, the p21 protein could inhibit the activity of the cell cycle-dependent proteinase compound (CyclinE-CDK2) to regulate the cell cycle and DNA repair and, ultimately, maintain the cells in the G1 stages for a prolonged period. In addition, the expression of the p53 protein could be regulated by interleukin-6 (IL-6), interleukin-17 (IL-17), interferon-*α* (INF-*α*), and other related cytokines. Such cytokines can bind to the corresponding receptors on the membrane, leading to the activation of aging-related signals by p53 [60].

**Figure 3:**
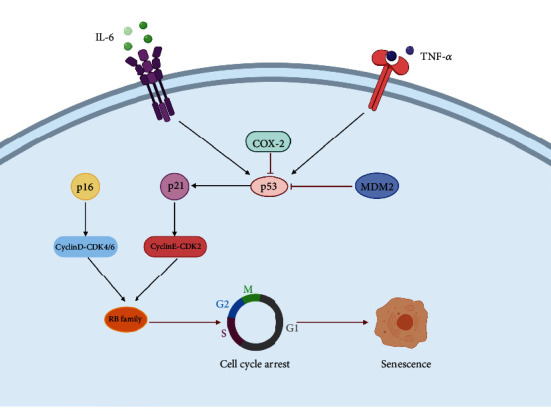
p53 promotes the progression of pulmonary fibrosis by inducing aging in lung fibroblasts. The expression of the p53 protein was regulated by multiple cytokines. COX-2 can inhibit the expression of p53. The cytokines IL-6 and TNF-*α* increased the expression of p53. In addition, murine double minute gene2 (MDM2) downregulated the expression of p53. The activation of p53 could induce p21, which then activated CyclinE-CDK2 to promote the expression of RB and initiate the cell aging pathway.

The effect of p53 on the regulation of lung fibroblast aging has been confirmed in a clinical study in which lung tissue was collected from patients with IPF and compared with tissues from healthy lung tissue. The results showed that the telomeres of lung fibroblasts in the lung tissue of patients with IPF were markedly shortened, and the expression of *β*-galactosidase was enhanced significantly, indicating obvious aging, in which the aging-related proteins p53, p21, and p16 played a crucial role [61]. Cyclooxygenase-2 (COX-2) is a specific upstream kinase of PGE2. The conventional belief is that COX-2 plays a proinflammatory role in pulmonary fibrosis. In contrast, some studies have shown that COX-2 primarily plays an anti-inflammatory role in pulmonary fibrosis. Hence, COX-2 plays a protective role in pulmonary fibrosis and can inhibit its progression [62, 63]. Our group found that when etoposide was added to MRC5 cells (human embryonic lung fibroblasts) to produce a model of aging, the expression of the p53 protein was markedly increased. A further study showed that the expression of the p53 protein was regulated by the COX-2 protein. COX-2 could delay the aging of lung fibroblasts through the inhibition of p53 protein expression [64]. Interleukin-18 (IL-18) was found to induce the aging of lung fibroblasts through the activation of the p53 protein, which accelerated the progression of pulmonary fibrosis. When IL-18 is inhibited, pulmonary fibrosis is effectively cured and p53-mediated lung fibroblast aging is effectively inhibited [65] (Figure 3).

### 4.3. Oxidative Stress

Oxidative stress is a strained state due to an imbalance between the oxidation and antioxidation systems [66]. When there is excessive reactive oxygen species (ROS) or insufficient antioxidants in the body, more ROS will be present in tissues or cells, which may trigger oxidative stress, leading to tissue or cell injury [67]. Oxidative stress is one of the pathogeneses of pulmonary fibrosis, which may play an important role in the progression of pulmonary fibrosis by promoting AEC necrosis, inducing epithelial cell apoptosis, regulating cytokine expression, and participating in EMT [68–70]. Moreover, specific oxidative injury in lung fibroblasts could also delay the progression of pulmonary fibrosis [71]. Previously, the design of drugs for the treatment of pulmonary fibrosis was concentrated mainly on the alleviation of AEC oxidative stress. The clinically representative medicine for the oxidative treatment of pulmonary fibrosis is N-acetyl-L-cysteine (NAC), which can prevent oxidative injury in AECs, thereby delaying the progression of pulmonary fibrosis [72]. Polyhexamethylene guanidine phosphate (PHMG-p) is a major component of disinfectants. PHMG-p can induce AEC injury and promote lung fibrosis. The addition of PHMG-p to A549 cells reportedly resulted in a significant increase in the level of apoptosis, as well as significant increases in the levels of ROS and P53 protein expression. Furthermore, after knockout of p53 in A549 cells and adding PHMG-p, the oxidative damage induced in the cells was significantly reduced [73]. This finding supports the hypothesis that the deleterious effects of PHMG-p on A549 cells may be related to the activation of redox reactions and P53 protein expression.

It was found that during the development and progression of pulmonary fibrosis, the expression of the p53 protein was positively correlated with the degree of oxidative stress (Table 2). The way in which p53 regulates the level of oxidative stress is still unclear. The effects of amitriptyline, an antidepressant, on pulmonary fibrosis treatment have been studied. The results showed that amitriptyline can inhibit the expression of nitric oxide synthase (iNOS), malondialdehyde (MDA), and lipid peroxides, and enhance the expression of glutathione (GSH), thereby alleviating the level of oxidative stress and delaying the progression of pulmonary fibrosis. Further studies have shown that the antioxidative stress of amitriptyline was probably related to its regulation of p53 [35].

**Figure 4:**
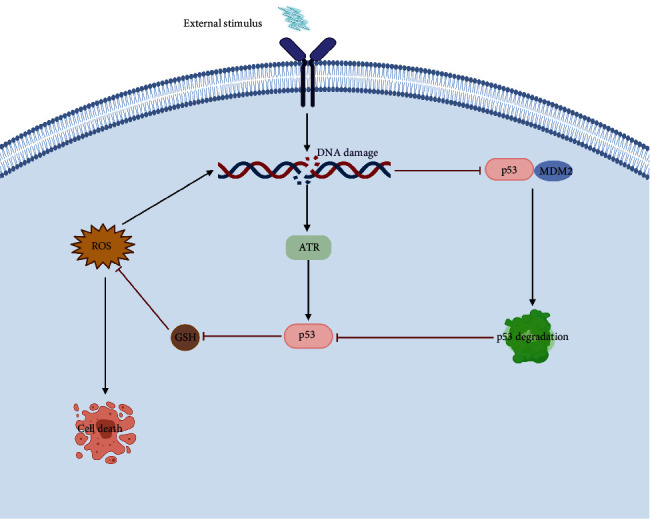
p53 promotes the progression of pulmonary fibrosis by enhancing the oxidative stress levels in AECs. In response to various external stimuli, the oxidant and antioxidant levels of AECs become unbalanced, leading to cellular DNA damage, which then transmits the signal downstream through various multiple pathways and initiates the oxidative stress system. First, DNA damage can activate the Rad3-related protein (ATR) and promote the expression of p53, which in turn reduces the expression of GSH and enhances the generation of intracellular ROS. The increase in ROS further aggravates DNA damage, leading to a vicious cycle. Second, DNA damage can interrupt ubiquitination, interfering with the binding of MDM2 to p53, which may reduce degradation of the p53 protein and promote its expression.

Given the broader understanding of p53, it was found that the p53 protein has the dual functions of oxidation and antioxidation, depending on the degree of oxidative stress [74]. In response to different degrees of oxidative stress, p53 may exert oxidative or antioxidative effects [75]. At present, most research supports the assumption that the enhanced expression of AECs in the early stages of pulmonary fibrosis could repair oxidation-induced AEC injury to a certain degree because of the mild degree of oxidative stress. The p53 protein enhanced the antioxidant capacity of cells by upregulating the expression of glutaminase-2 (GLS2) and increasing the levels of GSH and nicotinamide adenine dinucleotide (NADH). During the progression of disease, oxidative stress injury continues to increase, and the amount of ROS generated will enhance cellular injury, because the overexpression of the p53 protein can act directly on the promoter containing antioxidant response *cis*-elements (AREs) to inhibit nuclear factor E2-related factor 2- (Nrf2-) induced gene expression [76, 77] (Figure 4). Therefore, it is assumed that in the early stage of pulmonary fibrosis, when the degree of oxidative stress is very low, the p53 protein probably can function as a guide gene to repair AECs to a certain extent. As the disease progresses, ROS and other oxidative stress-related products accumulate to a certain degree, and the p53 protein accelerates the progression of pulmonary fibrosis.

### 4.4. EMT

Recent studies have shown the close correlation between EMT and the development and progression of pulmonary fibrosis [78]. In response to various stimuli, AEC may be converted to lung fibroblasts. As the number of lung fibroblasts increases, the degree of pulmonary fibrosis also increases [79]. Extensive study has shown that TGF-*β*1 was a key cytokine in the induction of EMT. TGF-*β*1 initially binds to the transmembrane serine/threonine receptor on the membrane of AEC, leading to the phosphorylation of upstream small mother against decapentaplegic2 (Smad2) or small mother against decapentaplegic3 (Smad3) at the carboxyl terminal, and is then translocated into the nucleus by forming a trimer with small mother against decapentaplegic4 (Smad4). After nuclear translocation, small mother against decapentaplegics (Smads) then bind to other transcription factors to regulate the expression of EMT-related genes, leading to the dysfunction of cellular junctions, cytoskeletal rearrangement, and enhancement of cell migration and cell invasion, and eventually to EMT [80].

Yamamoto et al. treated A549 cells with BLM and found that the morphology of A549 cells changed greatly. Meanwhile, the expression of alpha-smooth muscle actin (*α*-SMA), a marker protein for EMT, was significantly enhanced, suggesting that the EMT process had been initiated. Further studies found that p53 probably participated in the entire EMT process. BLM promoted the phosphorylation of Ser15 in the p53 protein, inhibiting the binding of MDM2, a negative regulator, to p53, and promoting the EMT process [81]. Wang et al. [47] presented a contrasting opinion. They induced the differentiation of A549 cells by the addition of different concentrations of TGF-*β*1, and found reduced expression of the p53 protein. It is assumed that p53 inhibits the process of EMT. The difference between studies is probably related to the following factors. Although the two models are *in vitro* EMT models, they are two different models. Direct disruption of A549 cells with BLM not only leads to EMT but also to pathological processes related to cell injury. However, it still remains unknown which process is dominant. In addition, both experiments were *in vitro* tests and could, therefore, not simulate the complete internal environment. Therefore, further experiments are required to validate the functional role of p53 in EMT.

## 5. Diagnosis and Treatment of Pulmonary Fibrosis

Currently, the diagnosis and assessment of pulmonary fibrosis rely mainly on a comprehensive analysis of the patient's medical history, clinical manifestations, high-resolution computed tomography (HRCT), and pulmonary function tests, as well as bronchoscopy or lung biopsy if necessary [82]. Serological testing is a valuable tool for disease screening and assessment. Serological tests are noninvasive, safe, and convenient. Unfortunately, in pulmonary fibrosis, there are no specific serologic tests for disease assessment [83]. Many clinical studies have demonstrated that the expression level of the serum p53 protein is positively correlated with the severity of pulmonary fibrosis. Therefore, it is conceivable that p53 could be a diagnostic or assessment indicator for the severity of pulmonary fibrosis. Currently, we believe that the diagnosis of pulmonary fibrosis should predominantly rely on computed tomography (CT) and pathology biopsies. Relying solely on serum p53 concentration for the diagnosis of pulmonary fibrosis is unjustified. Serum p53 levels can only be used as an auxiliary reference for the diagnosis of pulmonary fibrosis. Given the correlation of p53 with the severity of pulmonary fibrosis, the assessment of serum p53 expression to evaluate the changes in the condition of patients with confirmed pulmonary fibrosis may become a clinical reality in the future.

The establishment of a pulmonary fibrosis model by intratracheal instillation of BLM in p53 gene knockout and WT mice revealed significantly less collagen deposition in the lung tissue of p53 gene knockout mice and significantly reduced pulmonary fibrosis [36]. These observations suggested that the p53 gene knockout can slow the progression of pulmonary fibrosis. Several MDM2-p53 inhibitors are currently undergoing clinical trials as cancer treatments [84]. These trials provide some reference for drug therapy in pulmonary fibrosis. However, relevant to the pathogenesis of pulmonary fibrosis, the p53 protein has different underlying mechanisms of action on AECs and lung fibroblasts. Thus, the ideal drug would bidirectionally regulate these two types of cells through different mechanisms. Caveolin-1 scaffolding domain peptide (CSP) slows the progression of pulmonary fibrosis in mice by increasing the degradation of p53 by MDM2 and inhibiting p53 expression in AECs. In addition, unlike the reduced damage of AECs, CSP reduces the degradation of p53 by MDM2 in lung fibroblasts [36]. This in turn promotes p53 protein expression, which results in the increased apoptosis of lung fibroblasts and delayed progression of pulmonary fibrosis. However, such bidirectionally regulating drugs are relatively rare. Presently, the most feasible approach is targeted drug treatment with AECs or lung fibroblasts as the target. This approach will change the expression of p53 and slow the progression of pulmonary fibrosis.

## 6. Summary and Outlook

The weight of the evidence supports the hypothesis that the expression levels of p53 demonstrate a positive correlation with the severity of pulmonary fibrosis. However, p53 has various roles that are specific for different cells and have different mechanisms of action. During the progression of pulmonary fibrosis, an increase in p53 can promote AEC apoptosis, induce aging, aggravate oxidative injury, promote the progression of EMT, and accelerate the development of pulmonary fibrosis in terms of AECs. For lung fibroblasts, an increase in p53 can promote the apoptosis of lung fibroblasts, aggravate oxidative stress, and delay the development of pulmonary fibrosis. Meanwhile, increased p53 can also induce the aging of lung fibroblasts. This diversity makes it difficult to further clarify the regulatory mechanism of p53 in pulmonary fibrosis and to discover the corresponding drug treatment. Thus, for future treatment of pulmonary fibrosis, the targeted expression of p53 in different cells of lung tissues is a likely direction for study future studies which need to further focus on the following points.

The data from most of the current studies support the view that damage to AECs initiates pulmonary fibrosis and drives its development. The ultimate pathological change in pulmonary fibrosis is the massive proliferation of lung fibroblasts and copious secretion of collagen. Thus, is the damage to AECs or the proliferation of lung fibroblasts more important in the overall process of pulmonary fibrosis? Although AECs may be the most important, this view is speculative, with a dearth of valid confirmatory evidence.

p53 has different mechanisms of action for AECs and lung fibroblasts. This is a challenge for the development of drug therapies for pulmonary fibrosis that target p53. We believe that the current research on the underlying mechanisms of p53 in pulmonary fibrosis has been relatively superficial. Most studies have not addressed the function of the p53 protein. For example, the molecular mechanisms used by p53 to regulate AECs and lung fibroblasts are unknown. Furthermore, the p53 phosphorylation sites used to regulate apoptosis and senescence in AECs and lung fibroblasts are unknown. These issues must be clarified to further understand the role of p53 in the regulation of pulmonary fibrosis.

There have been few studies of drugs targeting p53 for the treatment of pulmonary fibrosis. However, many drugs that target p53 have been developed as cancer therapeutics. It is conceivable that these drugs may be beneficial for the treatment of pulmonary fibrosis.

Since p53 has different mechanisms of action in different cells, two points are crucial for the development of new drugs targeting p53 for the treatment of pulmonary fibrosis. First, we need to clarify whether the drug primarily targets AECs or lung fibroblasts. Second, we need to identify the signal transduction mechanism through which the drug exerts its effects. Targeting specific cells and specifically modulating p53 expression in those cells could achieve a better therapeutic outcome.

## Figures and Tables

**Table 1:** Studies evaluating p53 as a major regulator for apoptosis in pulmonary fibrosis.

Target	Mechanism	Model	Reference
p53	Apoptosis of AECs	AECs from BLM-induced pulmonary fibrosis in mice	[42]
p53, p21, Bcl-2	Proliferation and apoptosis of lung fibroblasts	BLM-induced pulmonary fibrosis in mice	[85]
p53	Apoptosis of AECs	BLM-induced pulmonary fibrosis in mice	[86]
p53, p21, Bcl-2, BAX	Apoptosis of AECs and lung fibroblasts	IPF patients	[43]
p53, BAX, caspase-9	Apoptosis of AECs	Asbestos was added to the cultivation medium of A549 cells	[87]
p53, p21, Bcl-2, BAX, Fas/Fasl	Apoptosis of AECs	BLM-induced pulmonary fibrosis in mice	[88]
p53	Apoptosis of AECs	BLM-induced pulmonary fibrosis in mice (iNOS^−/−^ and p53^−/−^ mice)	[89]
p53, p21, MDM2	Apoptosis of AECs, p53 degradation	IPF patients	[90]
p53, p21, Fas/Fasl	Apoptosis of AECs	IPF patients	[46]
p53, Bcl-2, BAX, Fas/Fasl, ROS	Apoptosis and oxidative stress of lung fibroblasts	Lung fibroblasts from BLM-induced pulmonary fibrosis in mice	[48]
p53, Bcl-2	Apoptosis of myofibroblasts	Pulmonary fibrosis patients, BLM-induced pulmonary fibrosis in rats	[91]
p53, E-cadherin	EMT of AECs, proliferation and apoptosis of lung fibroblasts	A549, MRC5	[47]
p53, MAPK	Apoptosis and oxidative stress of lung fibroblasts	Lung fibroblasts from BLM-induced pulmonary fibrosis in mice	[92]

**Table 2:** Studies evaluating p53 as a major regulator for senescence and oxidative stress in pulmonary fibrosis.

Target	Mechanism	Model	Reference
p53, p16, p12, COX-2	Senescence of AECs	BLM-induced pulmonary fibrosis in mice	[59]
p53, p21, IL-18	Senescence of lung fibroblasts	BLM-induced pulmonary fibrosis in mice, lung fibroblasts from BLM-induced pulmonary fibrosis in mice	[65]
p53, p21	Senescence of AECs	IPF patients	[93]
p53, p21	Senescence of AECs	BLM-induced pulmonary fibrosis in mice	[60]
p53, p16, p12, COX-2	Senescence of lung fibroblasts	Lung fibroblasts from BLM-induced pulmonary fibrosis in mice, MRC5	[64]
p53, p21, MDA, SOD	Oxidative stress of AECs	BLM-induced pulmonary fibrosis in mice	[94]
p53, IL-17A	Oxidative stress of AECs	BLM was added to the cultivation medium of A549 cells	[95]
p53	Oxidative stress of lung tissues	IPF patients	[96]
p53, iNOS, Nrf2, GSH	Oxidative stress of lung tissues	BLM-induced pulmonary fibrosis in mice	[35]

## Data Availability

The data used to support the findings of this study are available from the corresponding author upon request.

## Abbreviations

*α*-SMA: Alpha-smooth muscle actin
AEC: Alveolar epithelial cell
AECs: Alveolar epithelial cells
AECIIs: Type II alveolar epithelial cells
AREs: Antioxidant response *cis*-elements
ATR: Rad3-related protein
*β*-gal: *β*-Galactosidase
BM: Bone marrow
BLM: Bleomycin
CASP3: Caspase-3
CASP7: Caspase-7
CASP8: Caspase-8
COX-2: Cyclooxygenase-2
CSP: Caveolin-1 scaffolding domain peptide
CT: C terminus
CT: Computed tomography
EMT: Epithelial-mesenchymal transformation
GLS2: Glutaminase-2
GSH: Glutathione
HRCT: High-resolution computed tomography
IL-6: Interleukin-6
IL-17: Interleukin-17
IL-18: Interleukin-18
INF-*α*: Interferon-*α*
iNOS: Nitric oxide synthase
IPF: Idiopathic pulmonary fibrosis
MDA: Malondialdehyde
MDM2: Murine double minute gene2
NAC: N-Acetyl-L-cysteine
NADH: Nicotinamide adenine dinucleotide
NRF2: Nuclear factor E2-related factor 2
PDGF: Platelet-derived growth factor
PHMG-p: Polyhexamethylene guanidine phosphate
PRD: Proline-rich domain
ROS: Reactive oxygen species
SASP: Senescence-associated secretory phenotype
Smad2: Small mother against decapentaplegic2
Smad3: Small mother against decapentaplegic3
Smad4: Small mother against decapentaplegic4
Smads: Small mother against decapentaplegics
SNPs: Single nucleotide polymorphisms
SSCP: Single-strand conformation polymorphism
TAD: Transactivation domain
TD: Tetramerization domain
TGF-*β*1: Transforming growth factor-*β*1
TNF-*α*: Tumor necrosis factor-*α*
TUNEL: TdT-mediated dUTP nick-end labeling
WT: Wild type.

## References

[1] Sharif R. (2017). Overview of idiopathic pulmonary fibrosis (IPF) and evidence-based guidelines. *The American Journal of Managed Care*.

[2] Krauss E., Gehrken G., Drakopanagiotakis F. (2019). Clinical characteristics of patients with familial idiopathic pulmonary fibrosis (f-IPF). *BMC Pulmonary Medicine*.

[3] Lederer D. J., Martinez F. J. (2018). Idiopathic pulmonary fibrosis. *The New England Journal of Medicine*.

[4] Oldham J. M., Ma S. F., Martinez F. J. (2015). TOLLIP, MUC5B, and the response to N-acetylcysteine among individuals with idiopathic pulmonary fibrosis. *American Journal of Respiratory and Critical Care Medicine*.

[5] Richeldi L., Collard H. R., Jones M. G. (2017). Idiopathic pulmonary fibrosis. *Lancet*.

[6] Lee C. M., He C. H., Park J. W. (2019). Chitinase 1 regulates pulmonary fibrosis by modulating TGF-*β*/SMAD7 pathway via TGFBRAP1 and FOXO3. *Life Science Alliance*.

[7] Hou J., Ma T., Cao H. (2018). TNF-*α*-induced NF-*κ*B activation promotes myofibroblast differentiation of LR-MSCs and exacerbates bleomycin-induced pulmonary fibrosis. *Journal of Cellular Physiology*.

[8] Kishi M., Aono Y., Sato S. (2018). Blockade of platelet-derived growth factor receptor-*β*, not receptor-*α* ameliorates bleomycin-induced pulmonary fibrosis in mice. *PLoS One*.

[9] Wynn T. A. (2008). Cellular and molecular mechanisms of fibrosis. *The Journal of Pathology*.

[10] Zhang H. X., Li Y. N., Wang X. L. (2019). Probucol ameliorates EMT and lung fibrosis through restoration of SIRT3 expression. *Pulmonary Pharmacology & Therapeutics*.

[11] O’Dwyer D. N., Ashley S. L., Gurczynski S. J. (2019). Lung microbiota contribute to pulmonary inflammation and disease progression in pulmonary fibrosis. *American Journal of Respiratory and Critical Care Medicine*.

[12] Shi J., Zhou L. R., Wang X. S. (2018). KLF2 attenuates bleomycin-induced pulmonary fibrosis and inflammation with regulation of AP-1. *Biochemical and Biophysical Research Communications*.

[13] Nie Y., Sun L., Wu Y. (2017). AKT2 regulates pulmonary inflammation and fibrosis via modulating macrophage activation. *Journal of Immunology*.

[14] Maher T. M., Wells A. U., Laurent G. J. (2007). Idiopathic pulmonary fibrosis: multiple causes and multiple mechanisms?. *The European Respiratory Journal*.

[15] Lehmann M., Buhl L., Alsafadi H. N. (2018). Differential effects of Nintedanib and Pirfenidone on lung alveolar epithelial cell function in ex vivo murine and human lung tissue cultures of pulmonary fibrosis. *Respiratory Research*.

[16] Zhang Y., Guan L., Zheng Y., Mao L., Li S., Zhao J. (2019). Extracellular histones promote pulmonary fibrosis in patients with coal workers’ pneumoconiosis. *Journal of Occupational and Environmental Medicine*.

[17] Li Y., Gao Q., Xu K. (2018). Interleukin-37 attenuates bleomycin-induced pulmonary inflammation and fibrosis in mice. *Inflammation*.

[18] Chao C. C. (2015). Mechanisms of p53 degradation. *Clinica Chimica Acta*.

[19] Stein Y., Rotter V., Aloni-Grinstein R. (2019). Gain-of-function mutant p53: all the roads lead to tumorigenesis. *International Journal of Molecular Sciences*.

[20] Fu X., Wu S., Li B., Xu Y., Liu J. (2020). Functions of p53 in pluripotent stem cells. *Protein & Cell*.

[21] Kim E., Kim J. Y., Lee J. Y. (2019). Mathematical modeling of p53 pathways. *International Journal of Molecular Sciences*.

[22] Lacroix M., Riscal R., Arena G., Linares L. K., le Cam L. (2020). Metabolic functions of the tumor suppressor p53: implications in normal physiology, metabolic disorders, and cancer. *Molecular Metabolism*.

[23] Ho T., Tan B. X., Lane D. (2020). How the other half lives: what p53 does when it is not being a transcription factor. *International Journal of Molecular Sciences*.

[24] Brázda V., Fojta M. (2019). The rich world of p53 DNA binding targets: the role of DNA structure. *International Journal of Molecular Sciences*.

[25] Pfister N. T., Prives C. (2017). Transcriptional regulation by wild-type and cancer-related mutant forms of p53. *Cold Spring Harbor Perspectives in Medicine*.

[26] Liu L., Li D., Chen Z. (2017). Wild-type P53 induces sodium/iodide symporter expression allowing radioiodide therapy in anaplastic thyroid cancer. *Cellular Physiology and Biochemistry*.

[27] Kaur R. P., Vasudeva K., Kumar R., Munshi A. (2018). Role of p53 gene in breast cancer: focus on mutation spectrum and therapeutic strategies. *Current Pharmaceutical Design*.

[28] Liu M. C., Gelmann E. P. (2002). P53 gene mutations: case study of a clinical marker for solid tumors. *Seminars in Oncology*.

[29] Hojo S., Fujita J., Yamadori I. (1998). Heterogeneous point mutations of the p53 gene in pulmonary fibrosis. *The European Respiratory Journal*.

[30] LOK S. S., STEWART J. P., KELLY B. G., HASLETON P. S., EGAN J. J. (2001). Epstein-Barr virus and wild p53 in idiopathic pulmonary fibrosis. *Respiratory Medicine*.

[31] Korthagen N. M., van Moorsel C. H., Barlo N. P., Kazemier K. M., Ruven H. J. T., Grutters J. C. (2012). Association between variations in cell cycle genes and idiopathic pulmonary fibrosis. *PLoS One*.

[32] Liu T., De Los Santos F. G., Phan S. H. (2017). The bleomycin model of pulmonary fibrosis. *Methods in Molecular Biology*.

[33] Zhou Y., Li P., Duan J. X. (2017). Aucubin alleviates bleomycin-induced pulmonary fibrosis in a mouse model. *Inflammation*.

[34] Wu Q., Zhou Y., Zhou X. M. (2019). Citrus alkaline extract delayed the progression of pulmonary fibrosis by inhibiting p38/NF-*κ*B signaling pathway-induced cell apoptosis. *Evidence-based Complementary and Alternative Medicine*.

[35] Zaafan M. A., Haridy A. R., Abdelhamid A. M. (2019). Amitriptyline attenuates bleomycin-induced pulmonary fibrosis: modulation of the expression of NF-*κβ*, iNOS, and Nrf2. *Naunyn-Schmiedeberg's Archives of Pharmacology*.

[36] Nagaraja M. R., Tiwari N., Shetty S. K. (2018). p53 expression in lung fibroblasts is linked to mitigation of fibrotic lung remodeling. *The American Journal of Pathology*.

[37] Soysa N. S., Alles N. (2019). Positive and negative regulators of osteoclast apoptosis. *Bone Reports*.

[38] Abate M., Festa A., Falco M. (2020). Mitochondria as playmakers of apoptosis, autophagy and senescence. *Seminars in Cell & Developmental Biology*.

[39] Green D. R. (2019). Death by retrograde transport: avoiding the apoptosis default. *Cell Chemical Biology*.

[40] Larson-Casey J. L., Deshane J. S., Ryan A. J., Thannickal V. J., Carter A. B. (2016). Macrophage Akt1 kinase-mediated mitophagy modulates apoptosis resistance and pulmonary fibrosis. *Immunity*.

[41] Delbrel E., Soumare A., Naguez A. (2018). HIF-1*α* triggers ER stress and CHOP-mediated apoptosis in alveolar epithelial cells, a key event in pulmonary fibrosis. *Scientific Reports*.

[42] Bhandary Y. P., Shetty S. K., Marudamuthu A. S. (2013). Regulation of lung injury and fibrosis by p53-mediated changes in urokinase and plasminogen activator inhibitor-1. *The American Journal of Pathology*.

[43] Plataki M., Koutsopoulos A. V., Darivianaki K., Delides G., Siafakas N. M., Bouros D. (2005). Expression of apoptotic and antiapoptotic markers in epithelial cells in idiopathic pulmonary fibrosis. *Chest*.

[44] Rane C. K., Minden A. (2019). P21 activated kinase signaling in cancer. *Seminars in Cancer Biology*.

[45] Pérez-Yépez E. A., Saldívar-Cerón H. I., Villamar-Cruz O., Pérez-Plasencia C., Arias-Romero L. E. (2018). p21 activated kinase 1: nuclear activity and its role during DNA damage repair. *DNA Repair (Amst)*.

[46] Jinta T., Miyazaki Y., Kishi M. (2010). The pathogenesis of chronic hypersensitivity pneumonitis in common with idiopathic pulmonary fibrosis: expression of apoptotic markers. *American Journal of Clinical Pathology*.

[47] Wang M., Zhang J., Song X. (2013). Astaxanthin ameliorates lung fibrosis in vivo and in vitro by preventing transdifferentiation, inhibiting proliferation, and promoting apoptosis of activated cells. *Food and Chemical Toxicology*.

[48] Chuang C. Y., Liu H. C., Wu L. C., Chen C. Y., Chang J. T., Hsu S. L. (2010). Gallic acid induces apoptosis of lung fibroblasts via a reactive oxygen species-dependent ataxia telangiectasia mutated-p53 activation pathway. *Journal of Agricultural and Food Chemistry*.

[49] Soukas A. A., Hao H., Wu L. (2019). Metformin as anti-aging therapy: is it for everyone?. *Trends in Endocrinology and Metabolism*.

[50] Booth L. N., Brunet A. (2016). The aging epigenome. *Molecular Cell*.

[51] Colman R. J. (2018). Non-human primates as a model for aging. *Biochimica et Biophysica Acta - Molecular Basis of Disease*.

[52] Vanhooren V., Libert C. (2013). The mouse as a model organism in aging research: usefulness, pitfalls and possibilities. *Ageing Research Reviews*.

[53] Gerland L. M., Peyrol S., Lallemand C., Branche R., Magaud J. P., Ffrench M. (2003). Association of increased autophagic inclusions labeled for *β*-galactosidase with fibroblastic aging. *Experimental Gerontology*.

[54] Xia S., Zhang X., Zheng S. (2016). An update on inflamm-aging: mechanisms, prevention, and treatment. *Journal of Immunology Research*.

[55] Gulati S., Thannickal V. J. (2019). The aging lung and idiopathic pulmonary fibrosis. *The American Journal of the Medical Sciences*.

[56] Zank D. C., Bueno M., Mora A. L., Rojas M. (2018). Idiopathic pulmonary fibrosis: aging, mitochondrial dysfunction, and cellular bioenergetics. *Frontiers in Medicine*.

[57] Pardo A., Selman M. (2016). Lung fibroblasts, aging, and idiopathic pulmonary fibrosis. *Annals of the American Thoracic Society*.

[58] Wyman A. E., Noor Z., Fishelevich R. (2017). Sirtuin 7 is decreased in pulmonary fibrosis and regulates the fibrotic phenotype of lung fibroblasts. *American Journal of Physiology. Lung Cellular and Molecular Physiology*.

[59] Zhang C. Y., Duan J. X., Yang H. H. (2020). COX-2/sEH dual inhibitor PTUPB alleviates bleomycin-induced pulmonary fibrosis in mice via inhibiting senescence. *The FEBS Journal*.

[60] Lv X. X., Wang X. X., Li K. (2013). Rupatadine protects against pulmonary fibrosis by attenuating PAF-mediated senescence in rodents. *PLoS One*.

[61] Álvarez D., Cárdenes N., Sellarés J. (2017). IPF lung fibroblasts have a senescent phenotype. *American Journal of Physiology. Lung Cellular and Molecular Physiology*.

[62] Nakanishi T., Hasegawa Y., Mimura R. (2015). Prostaglandin transporter (PGT/SLCO2A1) protects the lung from bleomycin-induced fibrosis. *PLoS One*.

[63] Huang S. K., White E. S., Wettlaufer S. H. (2009). Prostaglandin E(2) induces fibroblast apoptosis by modulating multiple survival pathways. *The FASEB Journal*.

[64] Feng F., Wang Z., Li R. (2019). Citrus alkaline extracts prevent fibroblast senescence to ameliorate pulmonary fibrosis via activation of COX-2. *Biomedicine & Pharmacotherapy*.

[65] Zhang L. M., Zhang J., Zhang Y. (2019). Interleukin-18 promotes fibroblast senescence in pulmonary fibrosis through down-regulating Klotho expression. *Biomedicine & Pharmacotherapy*.

[66] Chainy G. B. N., Sahoo D. K. (2020). Hormones and oxidative stress: an overview. *Free Radical Research*.

[67] Yang S., Lian G. (2020). ROS and diseases: role in metabolism and energy supply. *Molecular and Cellular Biochemistry*.

[68] Hosseinzadeh A., Javad-Moosavi S. A., Reiter R. J., Yarahmadi R., Ghaznavi H., Mehrzadi S. (2018). Oxidative/nitrosative stress, autophagy and apoptosis as therapeutic targets of melatonin in idiopathic pulmonary fibrosis. *Expert Opinion on Therapeutic Targets*.

[69] Mirzaee S., Mansouri E., Shirani M., Zeinvand-Lorestani M., Khodayar M. J. (2019). Diosmin ameliorative effects on oxidative stress and fibrosis in paraquat-induced lung injury in mice. *Environmental Science and Pollution Research International*.

[70] Anathy V., Lahue K. G., Chapman D. G. (2018). Reducing protein oxidation reverses lung fibrosis. *Nature Medicine*.

[71] Yan B., Ma Z., Shi S. (2017). Sulforaphane prevents bleomycin-induced pulmonary fibrosis in mice by inhibiting oxidative stress via nuclear factor erythroid 2-related factor-2 activation. *Molecular Medicine Reports*.

[72] Huang H., Chen M., Liu F. (2019). N-Acetylcysteine tiherapeutically protects against pulmonary fibrosis in a mouse model of silicosis. *Bioscience Reports*.

[73] Park J. S., Park Y. J., Kim H. R., Chung K. H. (2019). Polyhexamethylene guanidine phosphate-induced ROS-mediated DNA damage caused cell cycle arrest and apoptosis in lung epithelial cells. *The Journal of Toxicological Sciences*.

[74] Liu X., Fan L., Lu C., Yin S., Hu H. (2020). Functional role of p53 in the regulation of chemical-induced oxidative stress. *Oxidative Medicine and Cellular Longevity*.

[75] Liu D., Xu Y. (2011). p53, oxidative stress, and aging. *Antioxidants & Redox Signaling*.

[76] Safdar A., Annis S., Kraytsberg Y. (2016). Amelioration of premature aging in mtDNA mutator mouse by exercise: the interplay of oxidative stress, PGC-1*α*, p53, and DNA damage. A hypothesis. *Current Opinion in Genetics & Development*.

[77] Beyfuss K., Hood D. A. (2018). A systematic review of p53 regulation of oxidative stress in skeletal muscle. *Redox Report*.

[78] Saito A., Horie M., Nagase T. (2018). TGF-*β* signaling in lung health and disease. *International Journal of Molecular Sciences*.

[79] Muthuramalingam K., Cho M., Kim Y. (2019). Cellular senescence and EMT crosstalk in bleomycin-induced pathogenesis of pulmonary fibrosis-an in vitro analysis. *Cell Biology International*.

[80] Margadant C., Sonnenberg A. (2010). Integrin-TGF-*β* crosstalk in fibrosis, cancer and wound healing. *EMBO Reports*.

[81] Yamamoto A., Kawami M., Konaka T., Takenaka S., Yumoto R., Takano M. (2019). Anticancer drug-induced epithelial-mesenchymal transition via p53/miR-34a axis in A549/ABCA3 cells. *Journal of Pharmacy & Pharmaceutical Sciences*.

[82] Saito S., Alkhatib A., Kolls J. K., Kondoh Y., Lasky J. A. (2019). Pharmacotherapy and adjunctive treatment for idiopathic pulmonary fibrosis (IPF). *Journal of Thoracic Disease*.

[83] Maher T. M. (2013). PROFILEing idiopathic pulmonary fibrosis: rethinking biomarker discovery. *European Respiratory Review*.

[84] Flemming A. (2016). Cancer: mutant p53 rescued by aggregation inhibitor. *Nature Reviews. Drug Discovery*.

[85] Pan Y., Fu H., Kong Q. (2014). Prevention of pulmonary fibrosis with salvianolic acid a by inducing fibroblast cell cycle arrest and promoting apoptosis. *Journal of Ethnopharmacology*.

[86] Bhandary Y. P., Shetty S. K., Marudamuthu A. S. (2012). Regulation of alveolar epithelial cell apoptosis and pulmonary fibrosis by coordinate expression of components of the fibrinolytic system. *American Journal of Physiology. Lung Cellular and Molecular Physiology*.

[87] Panduri V., Surapureddi S., Soberanes S., Weitzman S. A., Chandel N., Kamp D. W. (2006). P53 mediates amosite asbestos-induced alveolar epithelial cell mitochondria-regulated apoptosis. *American Journal of Respiratory Cell and Molecular Biology*.

[88] Kuwano K., Hagimoto N., Tanaka T. (2000). Expression of apoptosis-regulatory genes in epithelial cells in pulmonary fibrosis in mice. *The Journal of Pathology*.

[89] Davis D. W., Weidner D. A., Holian A., McConkey D. J. (2000). Nitric oxide-dependent activation of p53 suppresses bleomycin-induced apoptosis in the lung. *The Journal of Experimental Medicine*.

[90] Nakashima N., Kuwano K., Maeyama T. (2005). The p53-Mdm2 association in epithelial cells in idiopathic pulmonary fibrosis and non-specific interstitial pneumonia. *Journal of Clinical Pathology*.

[91] Zhang J., Xu P., Wang Y. (2015). Astaxanthin prevents pulmonary fibrosis by promoting myofibroblast apoptosis dependent on Drp1-mediated mitochondrial fission. *Journal of Cellular and Molecular Medicine*.

[92] Chen C. Y., Chen K. C., Yang T. Y., Liu H. C., Hsu S. L. (2013). Gallic acid induces a reactive oxygen species-provoked c-Jun NH2-terminal kinase-dependent apoptosis in lung fibroblasts. *Evidence-based Complementary and Alternative Medicine*.

[93] Jiang C., Liu G., Luckhardt T. (2017). Serpine 1 induces alveolar type II cell senescence through activating p53-p21-Rb pathway in fibrotic lung disease. *Aging Cell*.

[94] Su Z. Q., Liu Y. H., Guo H. Z. (2017). Effect-enhancing and toxicity-reducing activity of usnic acid in ascitic tumor-bearing mice treated with bleomycin. *International Immunopharmacology*.

[95] Gouda M. M., Prabhu A., Bhandary Y. P. (2018). Curcumin alleviates IL-17A-mediated p53-PAI-1 expression in bleomycin-induced alveolar basal epithelial cells. *Journal of Cellular Biochemistry*.

[96] Kusko R. L., Brothers J. F., Tedrow J. (2016). Integrated genomics reveals convergent transcriptomic networks underlying chronic obstructive pulmonary disease and idiopathic pulmonary fibrosis. *American Journal of Respiratory and Critical Care Medicine*.

